# Beyond the Grain: Navigating the Intersection of Gluten-Free Diets, Celiac Disease, and Cardiovascular Health

**DOI:** 10.7759/cureus.70430

**Published:** 2024-09-29

**Authors:** Miis Akel, William Delladio, Rosie Saikaly, Crystal Barroca, Paul Kaldas, Kelley L Davis, Marc M Kesselman

**Affiliations:** 1 Internal Medicine, Dr. Kiran C. Patel College of Osteopathic Medicine, Nova Southeastern University, Clearwater, USA; 2 Microbiology, Dr. Kiran C. Patel College of Allopathic Medicine, Nova Southeastern University, Fort Lauderdale, USA; 3 Rheumatology, Dr. Kiran C. Patel College of Osteopathic Medicine, Nova Southeastern University, Davie, USA

**Keywords:** cardiomyopathy, cardiovascular disease, celiac disease (cd), gluten-free diet (gfd), nutrition

## Abstract

Celiac disease (CD) is a complex autoimmune disorder triggered by gluten exposure in genetically susceptible individuals. This scoping review explores the intricate relationship between CD and cardiovascular manifestations, aiming to understand the impact of gluten-free diets (GFD) on cardiac health. A comprehensive search resulted in four pertinent studies revealing structural and functional abnormalities in the cardiovascular system of untreated CD patients. Among patients who did not adhere to a GFD, electrocardiogram abnormalities such as prolonged QT intervals and increased predisposition to arrhythmias were noted. Studies identified a significant prevalence of CD in patients with dilated cardiomyopathy or myocarditis, suggesting a potential association. GFD and immunosuppression were shown to improve cardiac function and reduce arrhythmias in CD patients exhibiting cardiovascular manifestations. However, limitations such as small sample sizes and incongruous cohorts make it difficult to assess the direct impact of GFD interventions on cardiac health. Future research should prioritize larger, longitudinal studies to fully understand mechanisms and treatment strategies. This review emphasizes the intricate interplay between CD and cardiac health, emphasizing the importance of early detection, comprehensive management, and further research for optimal clinical outcomes.

## Introduction and background

Celiac disease (CD) is an autoimmune disorder that reacts to gluten exposure in individuals with genetic susceptibility. This condition is associated with specific HLA genotypes, particularly HLA DR3-DQ2 and DR4-DQ8 [1]. CD onset occurs between 10 and 40 years of age, with a higher prevalence in females compared to males [2]. CD has a wide range of clinical presentations, including classic symptoms of diarrhea, steatorrhea, and weight loss, in addition to extraintestinal manifestations such as dermatitis herpetiformis [3]. The diagnosis of CD requires a comprehensive approach that combines endoscopy with duodenal biopsy to reveal villous atrophy. Serological markers, such as antibodies to tissue transglutaminase (IgA), endomysial IgA, and reticulin IgA in blood serum, help to confirm the diagnosis [4]. Additionally, individuals with type 1 diabetes, autoimmune thyroiditis, Down syndrome, and Turner syndrome are all categorized as high-risk individuals [5].

The pathogenesis of CD includes a multitude of immune events involving both adaptive and innate responses. When gliadin, a gluten-derived protein, is recognized by the immune system, chronic inflammatory cells infiltrate into the lamina propria and epithelium, eventually resulting in villous atrophy [6]. Gliadin-reactive T cells in the lamina propria recognize gliadin peptides bound to HLA DQ2 or DQ8 on antigen-presenting cells [6], resulting in immune dysregulation. Additionally, tissue transglutaminase catalyzes the deamidation of gluten peptides, which increases their immunogenicity [6]. In response to irritation and inflammation, endothelial cells and fibroblasts release tissue transglutaminase, which crosslinks glutamine-rich proteins such as gluten proteins, intensifying their binding to HLA-DQ2 and HLA-DQ8, thereby stimulating the T cell response [6]. In addition to the adaptive immune response, innate immunity plays an important role in promoting T cell-mediated immunity in CD, with food-derived triggers influencing intestinal epithelial cells and mononuclear cells. Interleukin 15 (IL-15) and type-I interferons (type-I IFNs) are cytokines produced by innate immune cells, and their production is heavily influenced by the gut microbiota. In the setting of a disrupted intestinal microenvironment, as seen in CD, these cytokines act on dendritic cells, intestinal epithelial cells, and intestinal epithelial lymphocytes, thus potentiating intestinal damage [7]. A strict gluten-free diet (GFD) remains the cornerstone of CD management, helping to alleviate symptoms and promote mucosal healing. The literature suggests a link between CD and cardiovascular manifestations such as autoimmune myocarditis or idiopathic dilated cardiomyopathy [8]. Notably, not all patients have classic gastrointestinal symptoms; iron deficiency anemia is a common clinical presentation.

A GFD is pivotal in CD management and shows promise in mitigating the cardiovascular complications associated with CD. Recent studies highlight the need for a more nuanced understanding of the impact of GFD on cardiovascular risk factors, emphasizing the interplay between dietary selections and cardiovascular well-being in individuals with and without CD. It is worth noting that CD can manifest in the heart in rare cases. Myocarditis, an extraintestinal manifestation of CD, occurs in 1.8%-5.7% of patients [8]. It is suspected that IgA antibodies can target the myocardium, as evidenced by their presence in untreated versus treated CD [8]. In those cases, a GFD is required to avoid additional cardiac complications such as dilated cardiomyopathy, heart failure, and arrhythmias [8,9]. Furthermore, CD has been linked to deteriorating arterial and aortic function due to inflammation and selenium deficiency, leading to increased aortic stiffness and stiffening of other major arteries, leading to atherosclerosis, coronary artery dissection, pericardial effusion, and their complications [8,9]. Initiating a GFD may be ineffective for those specific complications because restoring aortic elasticity is difficult; however, a GFD is useful in reversing ventricular dilation in the case of pericardial effusion. Moreover, deficiencies in protein S, folate, and vitamin B2 in patients with CD are linked to an increased risk of vascular thrombosis [8,10]. A GFD is helpful in decreasing the risk of thrombosis [8,10]. This review aims to explore the impact of GFD on cardiovascular manifestations of CD.

## Review

Methods

A systematic search of Embase was conducted to identify pertinent studies investigating the impact of GFD on cardiovascular outcomes and symptoms in patients with CD. Publications were considered up until January 31, 2024. The search strategy involved utilizing a combination of the keywords "gluten free diet" and "heart." This comprehensive approach aimed to capture a wide range of relevant literature on the subject.

The selection process encompassed cohort studies and randomized trials designed to provide valuable insights into the relationship between GFD and cardiovascular health in patients with celiac disease. Inclusion criteria involved studies with populations with diagnosed CD and the effects of a GFD on the outcomes of cardiovascular health. Studies that did not match the specific inquiry were excluded and specifically labeled as "Not Relevant" or "Incorrect Study Type." Systematic reviews, scoping reviews, meta-analysis reviews, and case reports were excluded under the "Incorrect Study Type" label for the purpose of this study.

**Figure 1:**
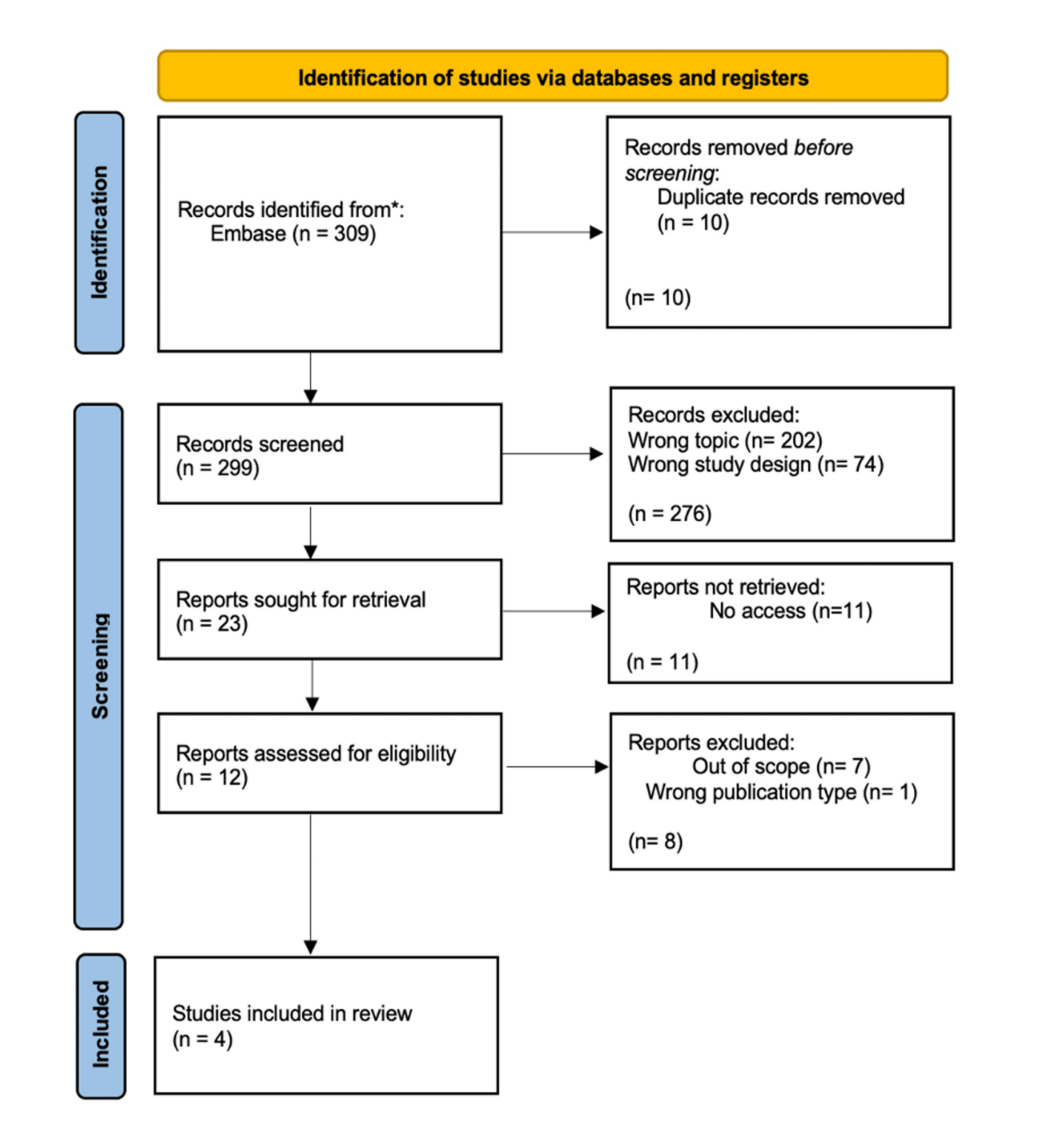
PRISMA flowchart: study screening and selection process. PRISMA: Preferred Reporting Items for Systematic Reviews and Meta-Analyses.

The subsequent data extraction process involved a thorough analysis of various elements, including study design, participant characteristics, dietary interventions, microbiota analysis methods, and nutritional outcomes. This meticulous approach was undertaken to ensure a comprehensive understanding of the methodologies employed and the outcomes reported in each study. A PRISMA (Preferred Reporting Items for Systematic Reviews and Meta-Analyses) study design was followed for this review (Figure 1).

The initial search criteria yielded 309 publications. Of those, 10 were excluded as duplicates, resulting in 299 studies. The resulting studies were initially screened for relevance to the topic. The initial screening resulted in the exclusion of 276 studies based on incorrect study type (74) or irrelevant topics (202). The following 23 papers were thoroughly reviewed based on the objective and inclusion/exclusion criteria. From the initially identified pool of studies, a total of four were ultimately chosen based on their adherence to the inclusion criteria, study type, and the completeness of available data. The selected studies were deemed to offer valuable contributions to the overall investigation into the impact of GFD on cardiovascular risk factors.

Results

Individuals diagnosed with CD exhibited a spectrum of structural cardiac anomalies, including mitral valve prolapse (MVP), minimal mitral regurgitation (MR), an enlarged left ventricular end-diastolic diameter, and often accompanied by a heightened prevalence of ejection fraction below 55% [11,12]. The investigations conducted by Sahin et al. and Bolia et al. emphasized the coexistence of both morphological and functional disruptions within the cardiovascular system of untreated CD patients [11,12].

Screening studies targeting individuals with dilated cardiomyopathy (DCM) or myocarditis revealed a significant prevalence of CD compared to control cohorts [13,14]. Among patients with DCM, symptomatology at the time of CD diagnosis varied, encompassing severe left ventricular dysfunction, recurrent abdominal pain associated with iron-deficiency anemia, and profound asthenia [13]. Interestingly, a subset of DCM patients with CD also presented with anemia, although exhibiting normal mean corpuscular volume (MCV) and serum ferritin concentration [13]. Among the patients with myocarditis, none of those with comorbid CD evaluated in this study exhibited typical CD-related gastrointestinal symptoms [14].

These investigations collectively supported a link between nutritional deficiencies resulting from chronic malabsorption in CD and the emergence of cardiomyopathy [11,13,14]. Notably, Frustaci et al. highlighted the universal presence of iron-deficiency anemia refractory to oral iron replacement among CD patients, further reinforcing this association [14]. Despite instances of folate deficiency, the reviewed studies did not identify atrial fibrillation in CD patients, thereby challenging previous assumptions [13].

**Figure 2:**
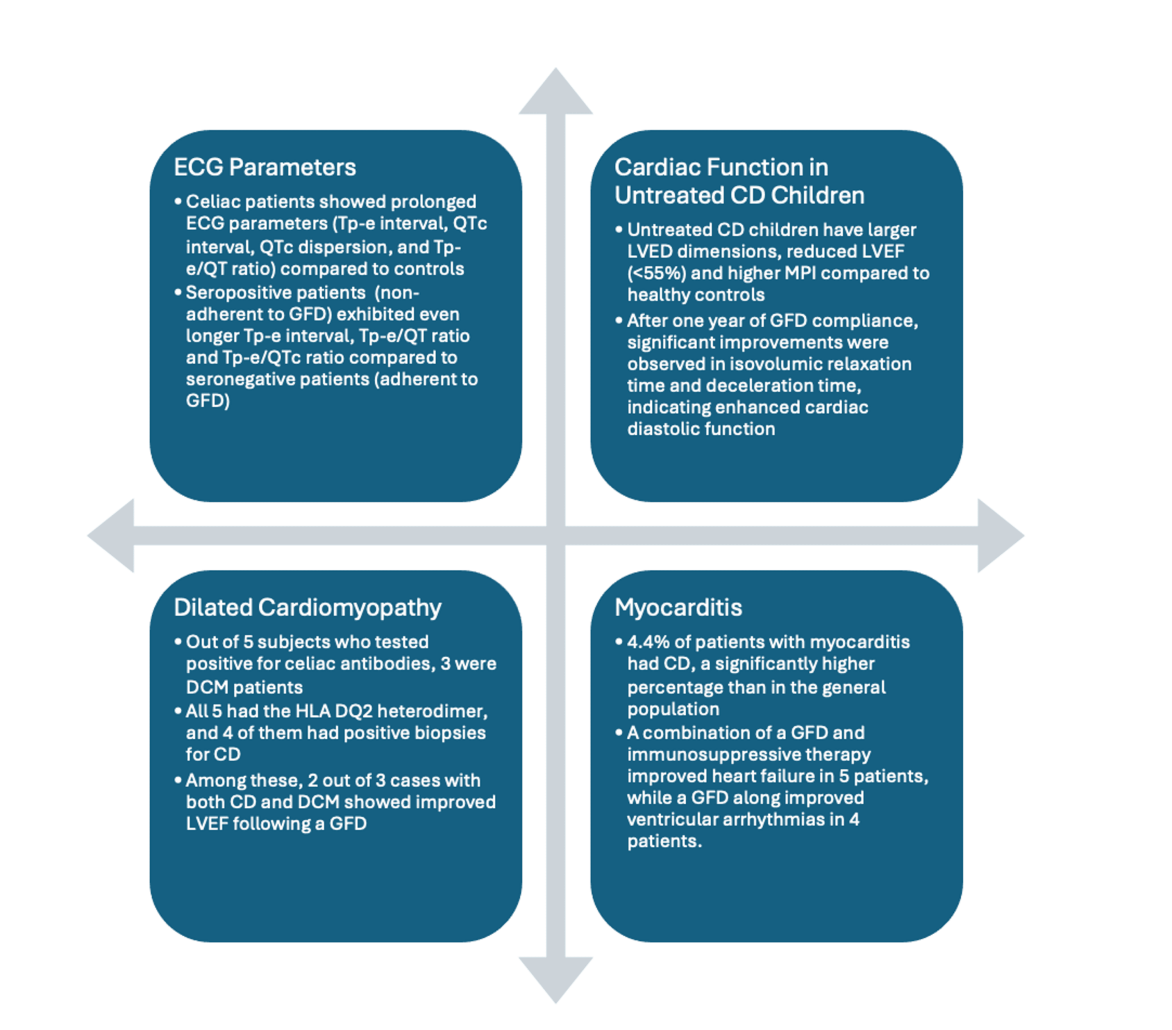
Diagram illustrating a summary of findings. ECG: electrocardiogram—a test that measures the electrical activity of the heart to show whether or not it is working normally. Tp-e: T-wave peak-to-end interval—the time between the peak and the end of the T-wave on an ECG, which may reflect the transmural dispersion of repolarization. QTc: corrected QT interval—the QT interval on an ECG, corrected for heart rate. It represents the time taken for the ventricles to depolarize and then repolarize. QTc dispersion: the difference between the maximum and minimum QTc intervals measured across different leads of the ECG. It indicates the variability in repolarization times within the myocardium. Tp-e/QT: the ratio of T-wave peak-to-end interval (Tp-e) to the QT interval. It is used as an index of ventricular repolarization. GFD: gluten-free diet—a diet that excludes gluten, a mixture of proteins found in wheat, barley, and rye, typically followed by individuals with celiac disease. CD: celiac disease—an autoimmune disorder where ingestion of gluten leads to damage in the small intestine. LVED: left ventricular end-diastolic diameter—a measurement of the size of the left ventricle of the heart at the end of the filling phase (diastole). LVEF: left ventricular ejection fraction—the percentage of blood that is ejected from the left ventricle during each heartbeat. It is a key indicator of heart function. DCM: dilated cardiomyopathy—a condition where the heart becomes enlarged and cannot pump blood effectively.

Electrocardiographic analyses revealed pronounced irregularities among CD patients, characterized by prolonged Tp-e interval (the interval from the peak of the T-wave to the end of the T-wave), corrected QT interval (QTc) (the QT interval is measured from the beginning of the QRS complex to the end of the T-wave; QTc is corrected according to the heart rate), QTc dispersion (the difference between the shortest and longest QTc intervals), and Tp-e/QT ratio (a marker of increased dispersion of ventricular arrhythmias) compared to control groups [11]. These studies established that individuals with CD experience changes in cardiac repolarization, leading to heightened susceptibility to ventricular arrhythmias (Figure 2) [11,14]. Notably, non-adherence to a strict GFD was associated with exacerbation of QT interval prolongation and the detection of ventricular arrhythmias [11]. Prolonged QTc and QTc dispersion in CD patients, relative to controls, were identified as potential indicators of ventricular arrhythmia risk [11,13].

These studies assessed the potential benefits of GFD [11-14] and GFD in combination with immunosuppressive therapies such as azathioprine and prednisone [14] in improving cardiac function and reducing arrhythmias in CD patients. Treatment results varied across studies, but the results suggest a correlation between these interventions and favorable CV response [11-14]. The association of GFD compliance with enhancements in left ventricular ejection fraction (LVEF), reduction in myocardial performance index (MPI), and improved cardiac diastolic function is significant [12]. Additionally, adherence to GFD was associated with improved cardiac diastolic function upon re-evaluation after one year, as evidenced by decreased isovolumic relaxation time and deceleration time [12]. Repetitive ventricular arrhythmias in one patient resolved with GFD, while symptoms such as tiredness improved in others, indicating the effectiveness of GFD [13]. Improvement in cardiac function was observed in patients treated with immunosuppression and GFD, accompanied by the disappearance of CD-specific autoantibodies following gluten withdrawal [14]. Moreover, Frustaci et al. emphasized the efficacy of GFD in alleviating cardiac arrhythmias, further accentuating its therapeutic value (Table 1) [14].

**Table 1:** Summary of the results and recommendations of the research articles. anti-tTG: anti-tissue transglutaminase; CD: celiac disease; DCM: dilated cardiomyopathy; ECG: electrocardiogram; ECHO: echocardiography; GFD: gluten-free diet; HF: heart failure; HLA-DQ2: human leukocyte antigen DQ2; IgA: immunoglobulin A; LVEF: left-ventricular ejection fraction; MPI: myocardial performance index; N: number; Tp-e: Tpeak-Tend; QTc: corrected QT.

Reference	Study Design	Data Collection	Study Aim	Findings	Recommendations	Limitations
Sahin et al. (2021) [11]	Longitudinal observational study	N= 61 subjects (33 control and 28 patients with CD). Echocardiography (ECHO) and 12-lead ECG were performed in all patients and controls.	To assess cardiac function and electrocardiogram (ECG) parameters associated with the development of atrial and ventricular arrhythmias in pediatric patients with celiac disease. Specifically, it investigates the differences in ECG parameters between celiac patients and healthy controls, as well as the impact of adherence to a gluten-free diet (GFD) on these parameters.	Several ECG parameters, including the Tp-e interval, QTc interval, QTc dispersion, and Tp-e/QT ratio, were significantly longer in celiac patients compared to the control group (p < 0.05). Additionally, when celiac patients were divided into seropositive (non-adherent to GFD) and seronegative groups (adherent to GFD), the seropositive patients exhibited even longer Tp-e interval, Tp-e/QT ratio, and Tp-e/QTc ratio compared to seronegative patients. The study also observed that untreated celiac patients, particularly those not adhering to a strict GFD, had a higher risk of ventricular arrhythmias, as indicated by the prolonged ECG parameters.	Further studies with larger cohorts and longer follow-up periods are recommended for a more comprehensive understanding of the relationship between celiac disease and cardiac arrhythmias in pediatric patients. Clinicians should pay attention to ECG parameters in CD patients to monitor for potential cardiac complications. The importance of strict adherence to GFD in managing celiac disease and potentially reducing the risk of cardiac arrhythmias.	Relatively small sample size The lack of long-term follow-up data. The study did not explore other potential confounding factors that might influence ECG parameters, such as medication usage or comorbidities.
Bolia et al. 2018 [12]	Longitudinal observational study	N= 175 participants, including 50 CD patients at diagnosis (group 1), 100 CD children in follow-up (group 2, comprising 47 compliant and 53 non-compliant patients), and 25 healthy controls.	To evaluate cardiac function in pediatric patients with celiac disease (CD) and investigate the impact of a gluten-free diet (GFD) on cardiac function. Specifically, the study aims to assess the prevalence of cardiac dysfunction in CD patients at diagnosis, observe changes in cardiac function after one year of GFD treatment in compliant patients, and compare the cardiac status of compliant and non-compliant CD patients.	Untreated CD children exhibited larger left ventricle end-diastolic dimension, reduced left ventricular ejection fraction (<55%), and higher myocardial performance index (MPI) compared to healthy controls. After one year of good dietary compliance, significant improvements were observed in isovolumic relaxation time and deceleration time, indicating improved cardiac diastolic function. Additionally, compliant patients showed lower MPI compared to non-compliant patients, indicating improvement in load-independent echocardiographic parameters.	The study suggests that subclinical cardiac dysfunction is common in pediatric CD patients at diagnosis. It highlights the importance of early diagnosis and initiation of GFD treatment to improve cardiac function in these patients. Additionally, the study emphasizes the significance of dietary compliance in maintaining cardiac health in CD patients.	A small sample size. The study duration of one year may not capture the long-term effects of GFD on cardiac function. The study did not investigate potential confounding factors such as comorbidities or medications that might influence cardiac function in CD patients.
Chicco et al. 2010 [13]	Cross-sectional observational study with some interventions	N=312 subjects (104 DCM patients, 44 first-degree relatives of the DCM patients, 101 healthy controls, 63 diseased controls).	To evaluate the practicality and accuracy of screening DCM patients for CD in cardiology clinics using a rapid assay.	5 total subjects tested positive for the antibodies, 3 of which were DCM patients (2.9% of DCM patients). All 5 had HLA DQ2 heterodimer. 4 of the 5 underwent biopsies, which were positive for CD. The subjects who tested negative on the rapid assay also tested negative on other diagnostic tests. 2 of the 3 cases with both CD and DCM had improved LVEF on a GFD.	Testing for IgA anti-tTG antibodies (rapid assay) is fast and reliable to diagnose CD in cardiology clinics. The rapid assay should be utilized in routine clinical exams.	Small sample size.
Frustaci et al. 2002 [14]	Retrospective observational study with some interventions	N=187 white Italian patients with myocarditis (118 males, 65 females); (110 admitted for HF, 77 admitted for arrhythmias).	To investigate the correlation between autoimmune myocarditis and CD.	4.4% of patients (patients) with myocarditis had CD (positive celiac panel and duodenal biopsies), significantly more than the general population. A combination of a GFD and immunosuppressive therapy improved HF in 5 patients. A GFD alone improved ventricular arrhythmias in 4 patients.	Patients with histologically significant myocarditis should be screened for CD.	The use of combination therapy of GFD and immunosuppressive therapy in a subset of their participants because of severe cardiac dysfunction causing a confounding variable.

Discussion

The findings from the reviewed studies provide detailed insights into the cardiac manifestations associated with CD, suggesting a potential link between CD and various structural and functional abnormalities in the cardiovascular system. Each study conducted rigorous comparisons between CD patients and control groups, meticulously controlling for demographic factors such as age, gender, height, and weight, thereby ensuring the comparability of study populations [11-14].

With cardiovascular disease being the leading cause of mortality worldwide [15] and CD widely thought to be a risk factor giving rise to several manifestations within the cardiovascular system, there is great interest in the nature and course of these cardiovascular pathologies when CD is appropriately diagnosed and treated. Thus, the goal of this scoping review was to identify available research demonstrating cardiovascular complications in patients with CD and examine the impact that GFD may have on these complications.

CD is an autoimmune enteropathy with an incidence estimated to be 1% worldwide [14]. It is mediated by intolerance to gluten protein, which is activated by tissue transglutaminase, causing a cascading effect by which CD4+ T cells induce cytokine release responsible for local small bowel mucosal changes, most notably villous atrophy and lymphocytic infiltration, and clinical manifestations, such as abdominal pain, diarrhea, and bloating [14]. Like many autoimmune disorders, recent findings reveal that CD has more systemic impacts than initially anticipated, including increased risk for cardiovascular and renal diseases when treatment is inadequate [16,17].

The association between CD and cardiovascular disease is not well understood; however, the leading theory suggests a multifactorial causation, including inflammatory pathways, endothelial dysfunction, and genetic susceptibility [18]. This complexity should not be understated. For both CD patients and the general population alike, increased age and white male populations carry an increased risk of developing coronary artery disease (CAD). In these CD-CAD patients, studies have shown an increased prevalence of hyperlipidemia [19]. Paradoxically, although this association has been elucidated between CD and risk of atherosclerotic disease, it is correlated with reduced incidence of classical risk factors of cardiovascular diseases, most notably lower BMI, total cholesterol, systolic blood pressure, and prevalence of type 2 diabetes mellitus [18]. While CD has traditionally been linked to a higher risk of cardiovascular disease, not all studies have found this same association. A recent retrospective case-control study by Dore et al. concluded that CD patients carry a reduced risk of developing cardiovascular disease [20]. Although these findings show some discrepancy with much of the research published on this topic, the authors presented a compelling assertion indicating that the reduced classical cardiovascular risk factors in the setting of CD from their study may stem from heightened attention to general health and wellness in those with CD. Therefore, this population may be less inclined to partake in behaviors such as smoking and a sedentary lifestyle that are considered modifiable risk factors [20]. This concept was not feasible to control based on their study design, but the contradictory findings between their study and prior research emphasized the complexity and paradoxical nature of the possible link between CD and cardiovascular diseases. With the exact nature of this relationship, continued effort in the research field toward understanding this link is warranted to help maximize patient outcomes and determine appropriate treatments.

One group of findings highlighted in our literature review was valvular abnormalities, most notably MVP and MR [11]. Valvular pathologies associated with CD are not well researched; therefore, the literature provides only a small glimpse into the nature of this association. Sahin et al. detected four trace incidences of mitral valve pathology in their cohort study through echocardiographic analysis, although the follow-up period of this study was unable to determine the effects of GFD on mitral valve health [11]. This warrants further evaluation to understand the potential role of GFD in modulating valvular insufficiencies. Ciaccio et al. highlighted a study by Cuoco et al. that demonstrated a high prevalence of MVP detected in patients with untreated CD [21]. Ciaccio et al. further highlighted the resolution of valvular regurgitations and improvement on echocardiogram in a study by Lionetti et al. [21]. However, this study only evaluated pediatric populations; thus, the scope of future research analyzing the potential link between valvular pathologies, CD, and the potential impact of GFD on these pathologies needs to be broadened to enhance our understanding of management in both pediatric and adult populations.

Another group of findings revealed structural abnormalities, most notably dilated cardiomyopathy, myocarditis [13,14], and increased left ventricular end-diastolic volume [11,12]. Several studies have reported an increased incidence of dilated cardiomyopathy in patients with CD [14,22]. Multiple cases have reported improvement in LVEF when celiac patients with dilated cardiomyopathy strictly adhere to GFD [23,24]. However, in a study by Milutinovic et al., the complex nature of GFD's impact on the heart should not be underestimated [22]. In the setting of strict adherence to GFD, histologic and endoscopic changes persist long after induction of the GFD [22], which emphasizes the importance of prompt initiation of GFD in patients with CD who develop DCM. Our literature review corroborates this message as patients adhering to strict GFD show improved LVEF and MPI [12]. Additionally, Frustaci et al. underscore this point in the setting of myocarditis. Given the aforementioned theory of chronic intestinal inflammation contributing to the insult on cardiac tissue, this prompt diagnosis and initiation of GFD is paramount. Despite the addition of cardioprotective medication, treatment may be suboptimal without effective control of CD at the level of the gastrointestinal mucosa due to potentiated inflammatory insult on cardiac tissue [23] and diminished absorptive capabilities [14].

The last group of findings highlighted in our review was conduction abnormalities, including arrhythmias. The effect of GFD in patients diagnosed with CD and conduction abnormalities has not been well-studied. However, cardiac conduction abnormalities have been documented in relation to systemic autoimmune diseases, including CD. A severe but rare known complication of CD in patients with poor GFD diet compliance is a celiac crisis, which can add a significantly increased risk for the development of arrhythmias as it is associated with severe dehydration and many electrolyte abnormalities, including hypokalemia, hypocalcemia, hypomagnesemia, and hypophosphatemia [25]. This failed compliance with GFD as a risk factor for celiac crisis adds support to the potential cardioprotective benefits of strict GFD.

In another study by Emilsson et al., a positive association between CD and atrial fibrillation was found, noting a 30% increased risk when patients with CD are compared to the general population [26]. This association may be attributed to the increased systemic inflammation, which was previously linked to a higher risk for atrial fibrillation [25]. Their study further supports the relationship between AF and CD, as discussed in the paper by Sahin et al. that was included in our scoping review [11,26]. Although Sahin et al. did not detect AF in their study population, it was indicated that the development of AF in patients with CD develops over an extended period of time as fibrotic changes affect the cardiac conduction pathways [11]. Both studies indicated that the relationship between the two conditions exists. The effect of a GFD on the association between CD and atrial fibrillation was not noted by Emilsson et al. However, it was mentioned that the association between AF and CD was greater in the patients with a more recent CD diagnosis [26]. This finding may point to a potential benefit in reducing the risk of cardiac conduction abnormalities in patients with CD who adhere to a GFD. Further research on the systemic effects of implementing a GFD in patients with CD is warranted.

The cardiac manifestations elucidated from our PRISMA search are not an all-encompassing list. Case reports have been published showing pericardial effusion in the setting of CD [27]. This case demonstrates an asymptomatic pericardial effusion found incidentally in an anemia workup. As seen in our review, iron-deficiency anemia is a common presentation associated with CD and the occurrence of other cardiovascular complications such as DCM [13,14]. Although the link between CD and pericardial effusion is not clear, initiation of GFD and iron supplementation in the case report showed improvement in peripheral edema and pericardial effusion [27]. However, iron supplementation is often not enough to correct the anemia in these patients. Due to villous atrophy from CD, strict adherence to GFD is imperative to these patients to stabilize the GI mucosa for adequate absorption of iron supplementation. The association between CD and pericardial effusion needs to be analyzed further to understand this relationship.

Additionally, our PRISMA search did not yield research showing the association between the development of congenital heart defects (CHDs) in infants born to mothers with CD. In a paper by Auger et al. analyzing over 2 million infants, they found that mothers with CD have a 1.58 times greater risk of having a baby with CHD when compared to mothers without CD [28]. This association is likely a product of inadequate CD control via GFD, leading to decreased absorption of crucial nutrients for the growth and development of the fetus in utero. However, there has been research published showing no association between mothers with CD and infants born with CHD as well. The association between CD and CHD is also shown in case reports of babies born with CHD who later develop CD [29]. This paper highlights the increased risk in the development of CD when trying to compensate for CHD-induced failure to thrive by adding gluten-containing cereals to the diet too early rather than emphasizing breast milk feeds. The relationship between CD and CHD is complex and not definitive; thus, further research is needed to understand this potential association.

The findings of this review strongly suggest that strict adherence to GFD can have cardioprotective benefits for patients with CD. It has been shown that there is a significantly higher risk of developing CD in patients who have a first-degree relative affected [30]. With genetic predisposition being a crucial factor in the development of CD, beginning in childhood, a thorough family history should be obtained from all patients in yearly physicals to identify at-risk patients. In patients with a strong family history, routine screening for CD should be considered by clinicians. If screening for CD is positive, routine screening for cardiovascular disease should be included in the treatment plan for these patients. As early CD can be asymptomatic, an insult to systemic tissues can begin years before typical gastrointestinal symptoms manifest. By emphasizing early detection and intervention in the CD patient population, we can minimize the systemic impacts associated with CD and optimize the health of these patients.

Limitations

Despite the valuable insights provided by the reviewed studies, several limitations were identified, including small sample sizes, heterogeneous patient populations, and limited follow-up durations. Future comprehensive research efforts should prioritize larger, longitudinal studies with homogeneous cohorts to elucidate the mechanisms and treatment strategies for cardiac manifestations in CD. Overall, the synthesis of findings underscores the intricate interplay between CD and cardiac health, highlighting the importance of early detection, comprehensive management, and further research to optimize clinical outcomes.

## Conclusions

Following a methodical search to understand the impact of a strict GFD on cardiovascular health, it is evident that patients with CD have an increased risk for cardiovascular complications, such as autoimmune myocarditis, dilated cardiomyopathy, arrhythmias, valvular diseases, and metabolic abnormalities, in comparison to the general population. Therefore, screening for cardiovascular diseases should be emphasized in CD patients for early detection and management. It can be concluded that a strict GFD can improve or prevent those cardiovascular complications. This improvement can be attributed to the reversal of certain nutritional deficiencies and a decrease in inflammatory processes. The exact pathophysiology linking CD to cardiovascular disease is not well understood, as is the mechanism by which a GFD might enhance cardiovascular health. Further research is warranted to dissect this correlation. Moreover, with the limitations of small sample sizes, limited follow-up durations, and the inability to measure the patients' strict adherence to a GFD, further research is needed that can address these study deficiencies.
